# Relationship Between the Use of Statins and Patient Survival in Colorectal Cancer: A Systematic Review and Meta-Analysis

**DOI:** 10.1371/journal.pone.0126944

**Published:** 2015-06-01

**Authors:** Heping Cai, Gongwu Zhang, Zhuo Wang, Zhihong Luo, Xiaochun Zhou

**Affiliations:** 1 Department of Pharmacy, Anhui Provincial Children's Hospital, Hefei, Anhui Province, China; 2 Department of Pharmacy, Changhai Hospital, Second Military Medical University, Shanghai, China; 3 Department of General Surgery, Lianyungang Oriental Hospital, Lianyun District, Lianyungang City, Jiangsu Province, China; Taipei Medicine University, TAIWAN

## Abstract

**Background:**

Studies have indicated that statins influence the risks and mortality rates of several types of solid tumors. However, the association between statin use and survival in patients with colorectal cancer (CRC) remains unclear.

**Methods:**

We searched the PubMed and Embase databases for relevant studies published up to September 2014 that assessed statin use and CRC prognosis. The primary outcomes were overall survival (OS) and cancer-specific survival (CSS). The secondary outcomes were disease-free survival (DFS) and recurrence-free survival (RFS). Hazard ratios (HRs) and 95% confidence intervals (CIs) were extracted and pooled with Mantel–Haenszel random-effect modeling. All statistical tests were two-sided.

**Results:**

Four studies on post-diagnosis statin therapy and five studies on pre-diagnosis statin use were included in our meta-analysis of 70,608 patients. Compared with the non-users, the patients with post-diagnosis statin use gained survival benefits for OS (HR 0.76; 95% CI: 0.68 to 0.85, P<0.001) and CSS (HR 0.70; 95% CI: 0.60 to 0.81, P<0.001). In addition, we observed that pre-diagnosis statin use prolonged the survival of patients with CRC for OS (HR 0.70; 95% CI: 0.54 to 0.91, P=0.007) and CSS (HR 0.80; 95% CI: 0.74 to 0.86, P<0.001). However, we did not observe a survival benefit for DFS (HR 1.13; 95% CI: 0.78 to 1.62, P=0.514) or RFS (HR 0.98; 95% CI: 0.36 to 2.70, P=0.975) in the CRC patients with post-diagnosis statin use.

**Conclusions:**

Statin use before or after cancer diagnosis is related to reductions in overall and cancer-specific mortality in colorectal cancer survivors.

## Introduction

As the most widely prescribed drugs for the treatment of hypercholesterolemia, statins or 3-hydroxy-3-methylglutaryl coenzyme A (HMG CoA) reductase inhibitors have been shown to reduce cardiovascular events and mortality in several randomized clinical trials [1–3]. In addition, they can prevent the progression of malignant cells[4] and modify their adhesive properties [5]. Indeed, several observational studies and meta-analyses have reported that statins are associated with reduced risks of several cancers, including primary liver [6], bladder [7], gastric [8], colorectal (CRC) [9], gynecologic [10], and lung cancers [11]. More recently, several studies have reported the effects of statins on the survival outcomes of solid cancers [12]. Several potential mechanisms have been reported regarding the effects of statins on cancers.

Statins could induce apoptosis by regulating several signaling pathways, including the MEK and ERK pathways [13]. Statins have been found to inhibit growth and induce apoptosis in CRC cell lines regardless of mutational status [14]. Statins were also found to influence angiogenesis and invasion by inhibiting geranylgeranylation of Rho family proteins [15]. Pravastatin has been reported to significantly inhibit colon carcinogenesis induced by the direct-acting carcinogen N-methyl-N-nitroso-urea in F344 rats [16].

Recent epidemiologic studies have shown reductions in mortality risk among statin users with ovarian [17], prostate [18], and renal cell [19] cancers and melanoma [20] compared with non-users.

Some studies over the last few years have indicated that statin use can improve survival in patients with CRC. However, the results are inconsistent. Lakha et al. [21] did not observe an association between overall survival (OS) or cancer-specific survival (CSS) and statin use post-CRC diagnosis or pre-CRC diagnosis. Cardwell et al. [22] reported that statin use in post-diagnosis CRC patients was associated with a 29% reduction in cancer-specific survival and that pre-diagnosis statin use was associated with a 14% reduction in cancer-specific survival.

To date, no systematic review has analyzed the association between statin use and prognosis in CRC patients. This research aimed to summarize published studies to gain a better understanding of the prognostic significance of statin use in CRC patients concerning OS, CSS, disease-free survival (DFS), and recurrence-free survival (RFS).

## Methods

### Data Sources and Searches

We performed a literature search using the PubMed and Embase databases for studies published up to September 2014. This literature search, which was performed using medical subject headings and Emtree headings and related text and keyword searches, was related to the prognostic effect of statin use on patients with CRC. The search terms were as follows: (‘colorectal neoplasms’ or ‘colon neoplasms’ or ‘rectal neoplasms’ or ‘large bowel neoplasms’) and (‘statin’ or ‘hydroxymethylglutaryl coenzyme reductase inhibitor’ or ‘HMG CoA reductase inhibitors’) and (‘survival’ or ‘prognosis’ or ‘mortality’). In addition, the references of all eligible studies were manually reviewed for additional relevant studies. Detailed search terms and strategies are provided in S1 File. The literature search was independently conducted by two authors (HPC and GWZ). The reference lists of the relevant articles were searched without language limitations.

### Study Selection and Eligibility Criteria

Two independent reviewers (HPC and GWZ) selected the identified studies by reading the titles and abstracts. The full-text version was retrieved for evaluation if the topic of the study could not be ascertained from its title or abstract. Discussions were performed with a third party (XCZ) to resolve any disagreements.

Studies were included if they met the following inclusion criteria: (1) the patients had colon or rectal cancer or CRC; (2) the association between statin use and prognosis was analyzed; (3) the time of statin use was before or after CRC diagnosis; and (4) hazard ratios (HRs) and 95% confidence intervals (CIs) were provided, or data were available that allowed for these variables to be calculated.

### Data Extraction

Two reviewers (HPC and GWZ) performed independent data extractions from eligible studies. Any disagreements were resolved by discussion with a third reviewer (ZHL) to reach a final consensus. We used a standardized data abstraction form to collect the following descriptive information: first author, country of origin, study design, tumor type, number of patients, stage, time at statin use onset (pre-diagnosis or post-diagnosis), treatment regimen, survival end points, adjusted variables and HR estimates with the corresponding 95% CIs of relevant outcomes.

### Assessment of Study Quality

The Newcastle-Ottawa scale (NOS) was used to evaluate methodological quality [23], which includes three main aspects of selection, comparability and exposure. A score of 5–8 points is considered high quality, and <5 points is considered low quality. Two authors (HPC and XCZ) independently performed the quality assessment. An additional author (ZW) examined and selected information independently according to the original studies.

### Statistical Analysis

We conducted statistical analyses using STATA statistical software (version 12.0; Stata Corporation, College Station, TX, USA). We applied DerSimonian and Laird random-effects models to pool the HRs for each analysis. All analyses were stratified by CRC diagnosis duration (statin use onset prior to or after CRC diagnosis). An HR greater than 1 indicated a worse prognosis in the statin user group, and an HR less than 1 indicated a better prognosis in the statin user group. OS was defined as the elapsed time between the date of CRC diagnosis and the date of death as a result of any cause. CSS was defined as the elapsed time between the date of CRC diagnosis and the date of death as result of CRC. In addition, we defined DFS as the time from the date of CRC diagnosis to that of tumor recurrence or the last follow at which no recurrence or metastasis was detected, and RFS was defined as the time from the date of CRC diagnosis to that of first tumor recurrence. The I^2^ statistic was used to assess heterogeneity by examining the percentage of interstudy variation, with values ranging from 0% to 100%. An I^2^ value less than 30% indicated no obvious heterogeneity, and a value greater than 50% was suggestive of increasing heterogeneity. Funnel plot asymmetry, as well as Begg’s rank correlation method and the Egger weighted regression method, were applied to explore potential publication bias, and a P value less than 0.05 was considered statistically significant. Sensitivity analysis was performed to investigate the influences of individual studies on the pooled effect size estimate by omitting one study at a time and recalculating the pooled estimate.

## Results

### Search Results

**Fig 1:**
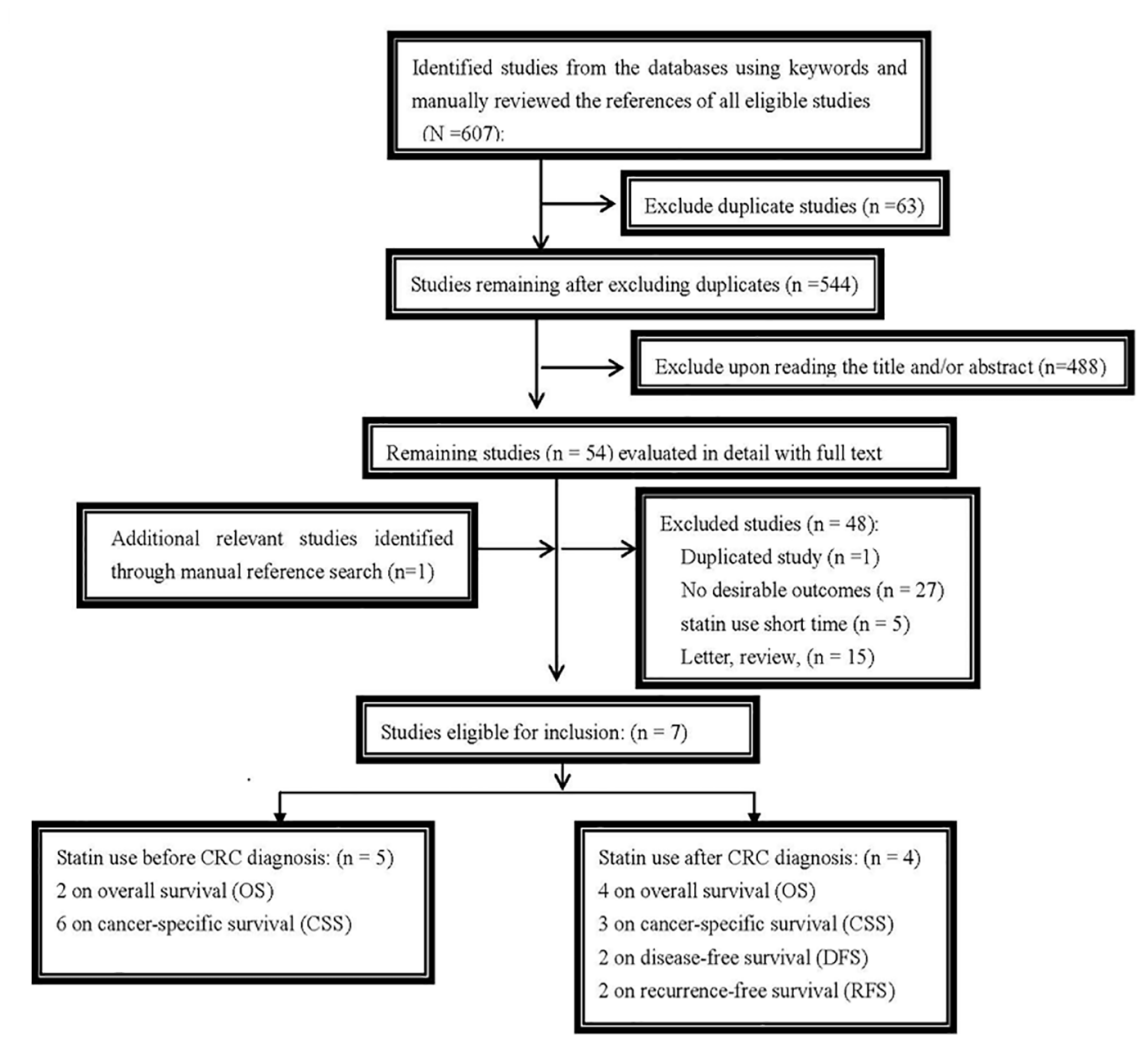
Flowchart of study selection.

A total of 607 potentially relevant articles were retrieved by our search strategy, and 54 studies remained after reviewing the titles and/or abstracts and elaborate reviews of the full texts; ultimately, seven studies met our inclusion criteria[12, 21, 22, 24–27]. These seven studies comprised 70,608 patients, four [21, 22, 26, 27] of which evaluated statin use after CRC diagnosis (all assessed OS, three investigated CRC-specific survival, two investigated DFS, and two investigated RFS), and five [12, 21, 22, 25, 26] of which assessed statin use before CRC diagnosis (two examined OS, and five reported CRC-specific survival). The detailed literature screening process is shown in Fig 1.

### Study Characteristics

The general characteristics of each study are listed in Table 1. The included studies consisted of five prospective studies [21, 22, 25–27] and two retrospective studies [12, 24], of which one was a nested case-control study [25], and six cohort studies [12, 21, 22, 24, 26, 27]. In accordance with the nine-point NOS, all included studies were of high quality, published after 2009 and applied multivariate analysis (S1 Table). Seven studies adjusted for age at diagnosis and sex. Four studies adjusted for drug combinations with aspirin or NSAIDs. Four studies included patients with CRC, two studies included those with colon cancer, and one included those with rectal cancer. The included studies assessed patients with different tumor stages. Three studies investigated patients with CRC of all stages (I-IV), one assessed stages I-III, one assessed stages I-II, one assessed stage III, and one did not report CRC stage. We next analyzed the follow-up times of these studies, of which five had a mean of over 5 years, one had a mean of 2.6 years and one did not report follow-up time. Three studies were performed in the USA, two studies in the UK, one in Denmark and one did not report the study location.

**Table 1:** Baseline characteristics of included studies in the meta-analysis.

First author (Year)	Origin country	Study design	tumor type	No. of patients	Stage	Pre/Post	Treatment regimen	Outcome	Adjusted variables
Cardwell, 2014	UK	prospective	CRC	14026	I-III	Pre,Post	S+chemo or radio	OS,CSS	year of diagnosis, age, sex, stage, surgery within 6 months, radiotherapy within 6 months, chemotherapy within 6 months, site, comorbidities, and other medication use after diagnosis as time-varying covariates, grade, deprivation, and smoking before diagnosis in individuals without missing values.
Mace,2013	USA	prospective	rectal	394	I-IV	Post	S+chemo or radio	OS,CSS, DFS,RFS	Age, BMI, ASA class III/IV, and pathological stage
Ma,2013	NR	retrospective	CRC	9950	I-II	Pre	S+chemo	OS,CSS	age, gender, stage, adjuvant therapy, co-morbidities, and the use of aspirin
Lakha,2012	UK	prospective	CRC	603	I-IV	Pre, Post	S+chemo or radio	OS,CSS	age, sex and AJCC stage
Nielsen,2012	Denmark	prospective	colon	43,487	NR	Pre	S+chemo or radio	CSS	age, stage, treatment with chemotherapy, treatment with radiotherapy, cardiovascular disease before cancer, diabetes mellitus before cancer, birth year, sex, descent, highest obtained level of education, and size of residential area
Ng,2011	USA	prospective	colon	839	III	Post	S+chemo	OS,DFS,RFS	age, sex, family history of colorectal cancer, baseline performance status, depth of invasion through bowel wall, number of positive lymph nodes, perineural invasion, extravascular invasion, postoperative carcinoembryonic antigen, treatment arm, body mass index, physical activity, Western pattern diet, and consistent aspirin use.
Siddiqui,2009	USA	retrospective	CRC	1309	I-IV	Pre	S+chemo or radio	CSS	BMI and the use of NSAIDs

Abbreviations: NR, not reported; Pre, prediagnosis; Post, postdiagnosis; S, surgery; chemo, chemotherapy; OS, overall survival; CSS, cancer-specific survival; DFS, disease-free survival; RFS, recurrence-free survival; BMI, Body Mass Index; NSAIDs; Non-steroidal anti-inflammatory drugs; AJCC, American Joint Committee on Cancer; ACEIs, angiotensin converting enzyme inhibitors;

### Quantitative Data Synthesis

#### Statin use before and after CRC diagnosis and survival

**Fig 2:**
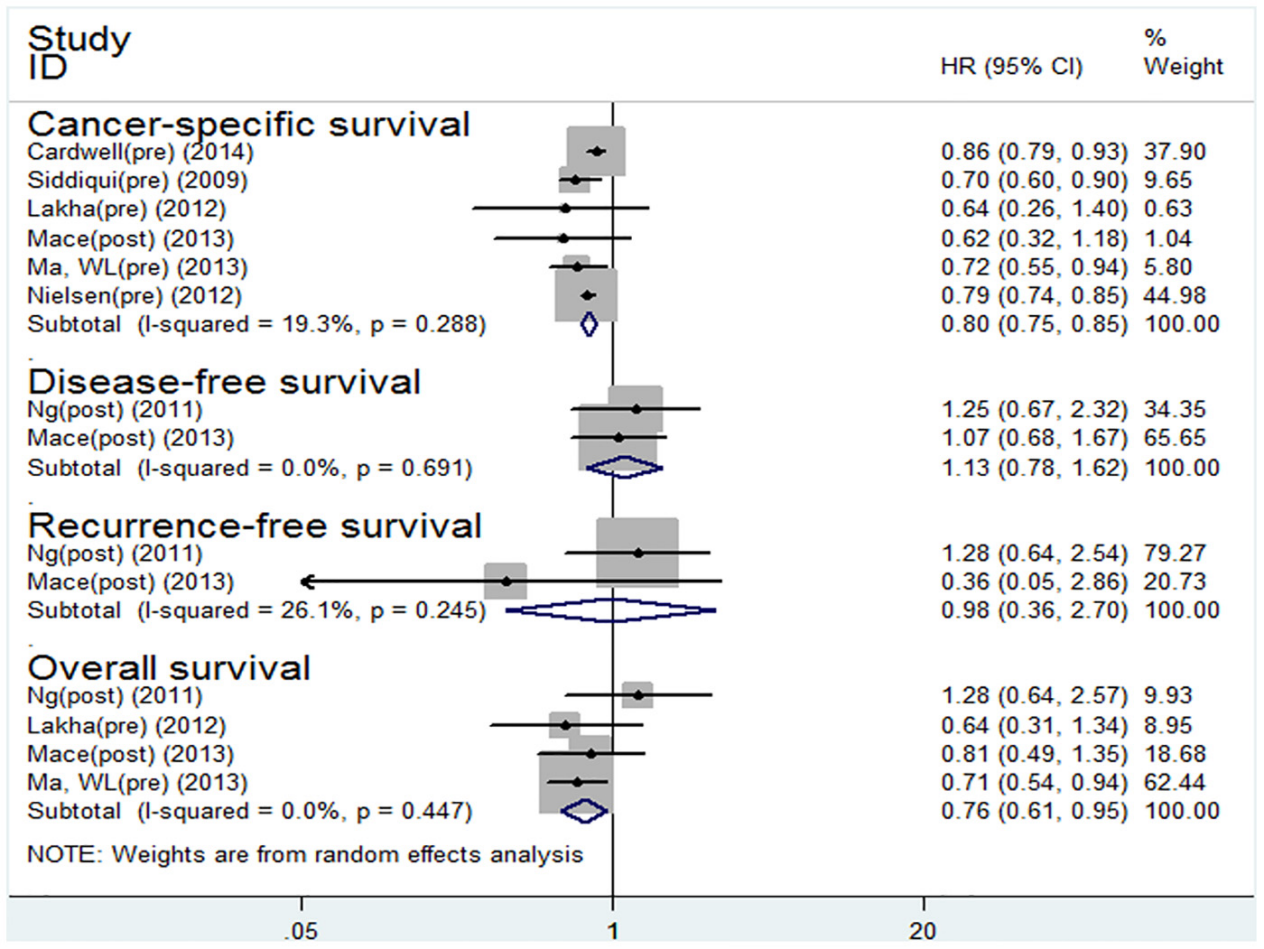
Forest plot of statins use and patient survival.

Seven studies with 70,608 patients on statin therapy (pre-diagnosis and post-diagnosis) were included in our meta-analysis. Compared with the non-users, the patients using statins gained survival benefits for OS (HR 0.76; 95% CI: 0.61 to 0.95, P = 0.016) and CSS (HR 0.80; 95% CI: 0.75 to 0.85, P<0.001) (Fig 2).

#### Statin use after colorectal cancer diagnosis and survival

Four studies on post-diagnosis statin therapy were included in our meta-analysis. Compared with the non-users, the patients with post-diagnosis statin use gained survival benefits for OS (HR 0.76; 95% CI: 0.68 to 0.85, P<0.001) and CSS (HR 0.70; 95% CI: 0.60 to 0.81, P<0.001).

**Fig 3:**
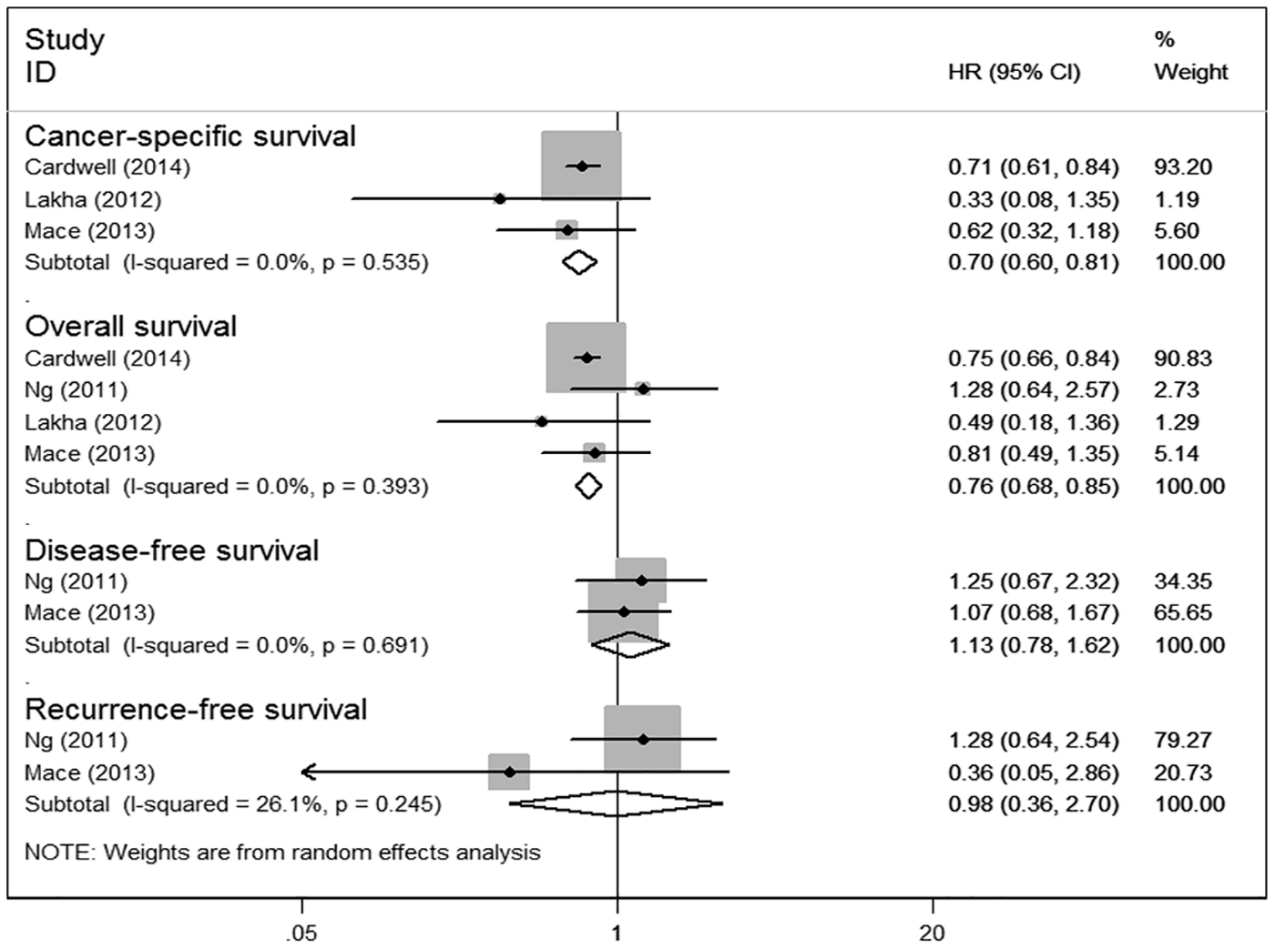
Forest plot of statins use after diagnosis of colorectal cancer and patient survival.

However, no survival benefit was observed for DFS (HR 1.13; 95% CI: 0.78 to 1.62, P = 0.514) or RFS (HR 0.98; 95% CI: 0.36 to 2.70, P = 0.975) in the CRC patients with post-diagnosis statin use (Fig 3).

**Fig 4:**
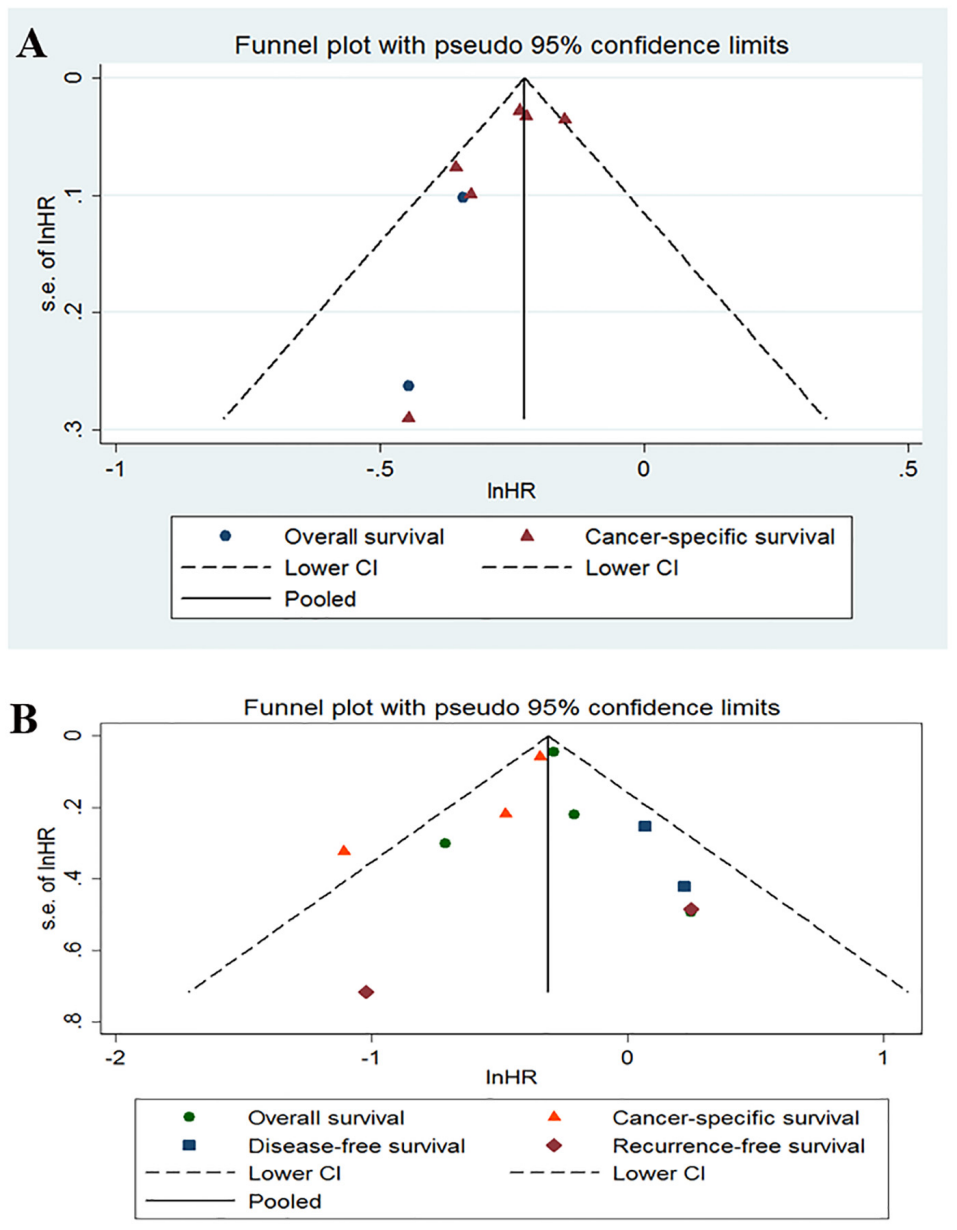
Funnel of statins use before and after diagnosis of colorectal cancer and patient survival.

A funnel plot for publication bias assessment is illustrated in Fig 4.

#### Statin use before CRC diagnosis and survival

**Fig 5:**
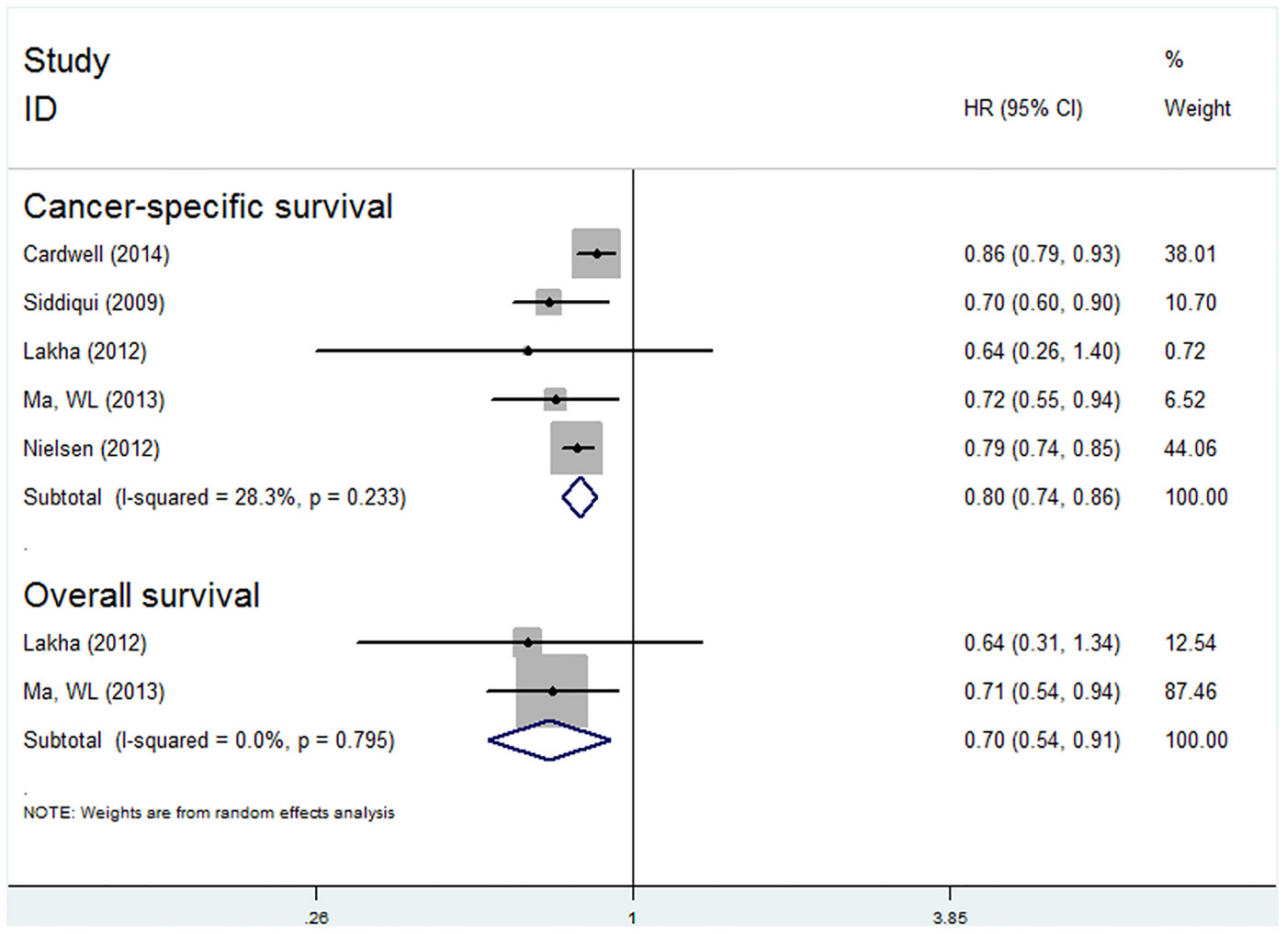
Forest plot of statins use before diagnosis of colorectal cancer and patient survival.

Five studies on pre-diagnosis statin use were included in our meta-analysis. Compared with the non-users, we observed that pre-diagnosis statin use prolonged the survival of CRC patients for OS (HR 0.70; 95% CI: 0.54 to 0.91, P = 0.007) and CSS (HR 0.80; 95% CI: 0.74 to 0.86, P<0.001) (Fig 5).

#### Statin use after rectal cancer diagnosis and survival

**Fig 6:**
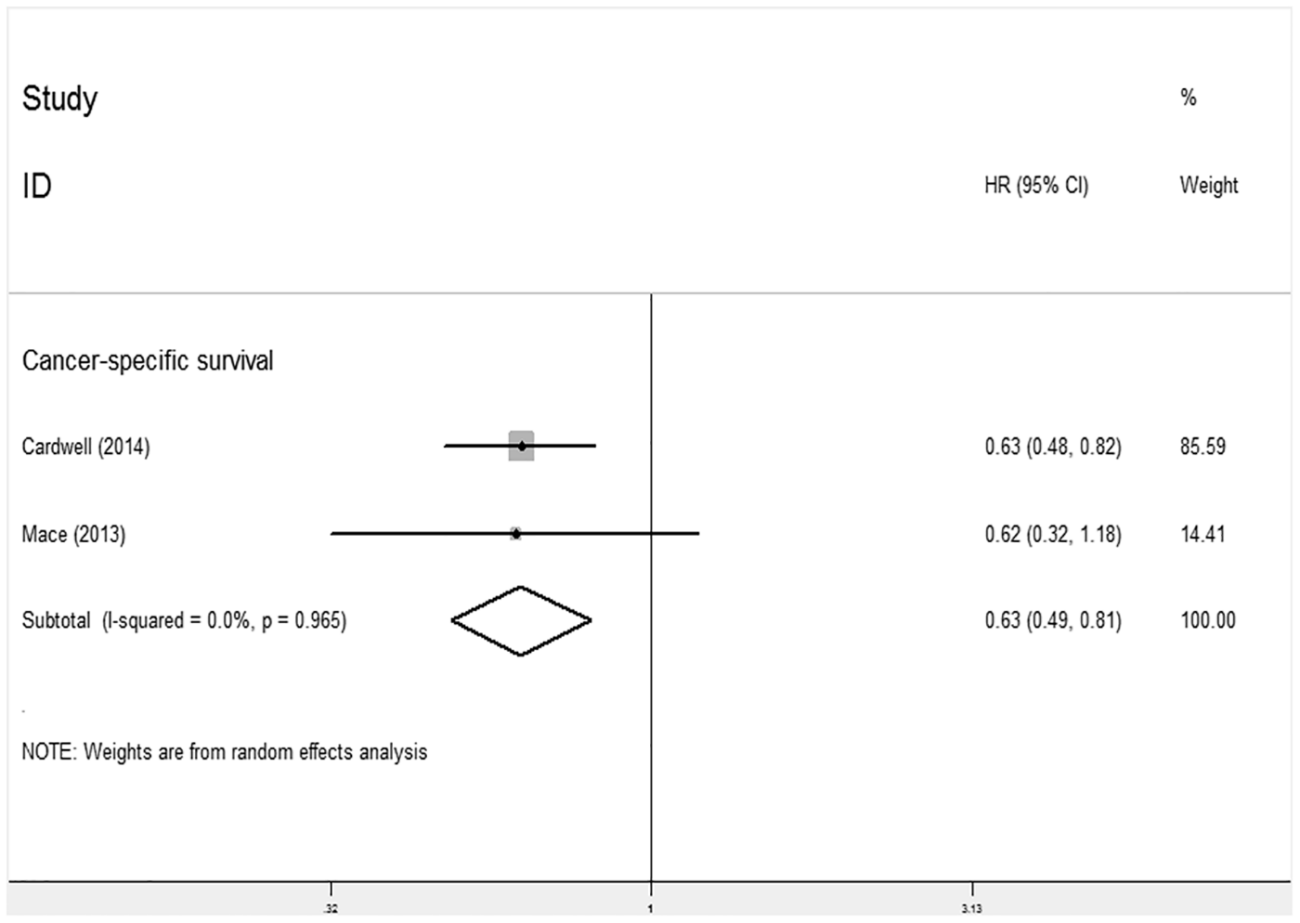
Forest plot of statins use after diagnosis of rectal cancer and patient survival.

Two studies investigated the relationship between post-diagnosis statin use and CSS for rectal cancer and indicated that statin use post-diagnosis was significantly associated with CSS for this type of cancer (HR 0.63; 95% CI: 0.49 to 0.81, P<0.001) (Fig 6). Due to the limited number of studies, we did not analyze the relationship between post-diagnosis statin use and CSS for colon cancer.

### Sensitivity Analysis

Sensitivity analysis was conducted in the OS subgroup by omitting each study and recalculating the pooled HR estimates (HR 0.75; 95% CI: 0.65 to 0.86, P<0.001), which did not significantly alter the pooled HRs.

### Publication Bias

Funnel plots (Fig 4) showed that there was no evident asymmetry for prediagnosis statin use and postdiagnosis statin use. No significant bias was detected using Begg’s regression analysis (P = 0.734) for OS or CSS (P = 0.296) and Egger’s test for OS (P = 0.970) or CSS (P = 0.310).

## Discussion

In this study, which evaluated seven observational studies with 70,608 patients, we found that statin use was associated with reduced overall mortality and CRC-specific mortality. Analyses stratified by statin use before and after CRC diagnosis showed that post-diagnosis statin use led to a 30% reduction in CRC-specific mortality and a 24% reduction in overall mortality compared with non-users. However, our findings showed that pre-diagnosis statin use led to a 20% reduction in CRC-specific mortality and a 30% reduction in overall mortality compared with non-users. However, post-diagnosis statin use did not improve DFS or RFS.

Although our results are relatively consistent, there are several limitations in this meta-analysis due to the nature of observational studies. First, despite the fact that nearly all studies adjusted for some potential confounders, we cannot eliminate the possibility that the final associations or results are confounded by some other unmeasured variables, such as a history of taking additional medication, including hypoglycemic agents and hypotensive drugs, lifestyle and molecular features of the tumors. Furthermore, the measured confounding variables (such as age, sex, and disease stage) were possible but not definitive causes of the observed strong associations between statin use and survival outcome. We did not conduct subgroup analyses because of the limited number of studies and insufficient data available. Therefore, we did not investigate other factors affecting prognosis, such as microsatellite instability (MSI) [28], CRC patients with ulcerative colitis (UC) [29], the impact of anastomotic leakage after surgery on colorectal cancer (CRC) [30], the chromosome 18q genotype [31], DCC status[31], etc.

Second, we did not explore the impact of drug combinations on survival outcomes because several included studies reported that patients took not only statins but also aspirin or other non-steroidal anti-inflammatory drugs (NSAIDs). Siddiqui et al. (2009) [12] found no evidence of interactions between statins and confounding factors, such as the use of aspirin/NSAIDs in CRC patients, and also found no synergistic effects of the use of statins combined with aspirin/NSAIDs on CRC development [32, 33]. Ma et al. (2013) [27] indicated that patients who took statins in addition to aspirin before diagnosis had improved OS and CSS. However, Ng et al. (2009) [25] showed that those who took statins and aspirin after diagnosis did not have improved OS or CSS. Thus, whether drug combinations impact CRC outcome requires further study.

Third, this meta-analysis did not further analyze the effects of the type, dose and duration of statins on the prognosis of CRC patients because no detailed information was provided in the studies that could have resulted in less accurate estimations of prognosis for statin therapy. Lakha et al. (2012) [21] found no significant association between statin dosage and CSS in CRC patients. In addition, Nielsen et al. (2012) [24] found that drug dosage was not significantly associated with CSS. Kaye et al. [34] found that statin use for more than 3 months was not significantly associated with CRC. We suggest that statin use for three months may not be sufficient to detect differences, and different statins may influence prognosis. Previous studies have not found that the use of different statins impacts CRC risk [26], but further study is necessary.

Fourth, the included studies were primarily observational and might have been affected by selection bias. Publication bias also may be inevitable in meta-analysis because some studies with insignificant [35] results or negative outcomes are unlikely to be published. Although we detected symmetry in the funnel plots of Egger’s test in our study, as a result of the limited number of included studies, we did not confirm whether publication bias existed in the current meta-analysis. Thus, inadequate control of the confounders might have led to an exaggeration or underestimation of the survival benefit estimates.

This study has several strengths. First, all previous studies focused on the relationship between statin use and the risk of CRC [36, 37]. Our study is the first meta-analysis with a large sample size of more than 75,000 patients assessing the relationship between statin use and CRC prognosis that provides adequate power to detect differences in mortality attributed to statin use and CRC prognosis. Second, we used an exhaustive and reproducible search strategy to retrieve all relevant literature in PubMed and Embase databases. Third, we conducted sensitivity analysis of the association between post-diagnosis statin use for OS, and the results were robust. In addition, we abstracted the survival data of multivariate Cox proportional hazard models, which greatly reduced the influence of confounding bias.

In summary, our findings based on this meta-analysis demonstrate that statin use before and after CRC diagnosis is associated with improved OS and CRC-specific survival in patients with CRC. The improved CSS with statin use persists in patients with rectal cancer. Large, prospective, randomized trials should be conducted to investigate the association between the effects of statin dose and type on CRC survival.

## Supporting Information

S1 File Search Phrases for a) Pubmed, and b) Embase. (DOCX)

S1 Table Quality assessment of the included studies. (DOCX)

S1 Checklist PRISMA Checklist. (DOC)
