# Supplementary material for: Relationship Between the Use of Statins and Patient Survival in Colorectal Cancer: A Systematic Review and Meta-Analysis
Source: PLoS One. 2015 Jun 1;10(6):e0126944. doi: 10.1371/journal.pone.0126944 (PMC4451009; doi:10.1371/journal.pone.0126944)
Supplement: S1 File — (DOCX) [file pone.0126944.s001.docx]

**Supporting Information**

**S1 File.** Search Phrases for a) Pubmed, and b) Embase

Search included:

Pubmed to September 2014

Embase to September 2014

**a) Pubmed： Results 125**

Search (((((((("Colonic Neoplasms"[Mesh]) OR "Rectal Neoplasms"[Mesh]) OR "Colorectal Neoplasms"[Mesh])) OR ((((((((((colon[Title/Abstract]) OR colonic[Title/Abstract]) OR rectal[Title/Abstract]) OR rectum[Title/Abstract]) OR colorect*[Title/Abstract]) OR large bowel[Title/Abstract])) AND ((((((cancer*[Title/Abstract]) OR tumor*[Title/Abstract]) OR tumour*[Title/Abstract]) OR carcinoma*[Title/Abstract]) OR neoplas*[Title/Abstract]) OR malignan*[Title/Abstract])))) AND (("Hydroxymethylglutaryl-CoA Reductase Inhibitors"[Mesh]) OR ((((((((hydroxymethylglutaryl-coenzyme A reductase inhibitor[Title/Abstract]) OR statin*[Title/Abstract]) OR fluvastatin[Title/Abstract]) OR pravastatin[Title/Abstract]) OR lovastatin[Title/Abstract]) OR simvastatin[Title/Abstract]) OR atorvastatin[Title/Abstract]) OR rosuvastatin[Title/Abstract]))) AND (((("Survival"[Mesh]) OR "Mortality"[Mesh]) OR "Prognosis"[Mesh]) OR ((((((Prognos*[Title/Abstract]) OR outcome*[Title/Abstract]) OR survival[Title/Abstract]) OR mortality[Title/Abstract]) OR recurren*[Title/Abstract]) OR predict*[Title/Abstract]))

**b) Embase： Results 482**

#1 'colon cancer' 167870

#2 'rectum cancer' 127726

#3 'colorectal cancer' 85298

#4 #1 OR #2 OR #3 187949

#5 ((colon OR colonic OR rectal OR rectum

OR colorect* OR (large AND bowel))AND (cancer* OR tumor* OR tumour*

OR carcinoma* OR neoplas* OR malignan*) 103673

#6 #4 OR #5 28684

#7 'hydroxymethylglutaryl coenzyme a reductase inhibitor' 95970

#8 'hydroxymethylglutaryl-co a reductase inhibitor'

OR statin* OR fluvastatin OR pravastatin OR lovastatin

OR simvastatin OR atorvastatin OR rosuvastatin 50113

#9 #7 OR #8 104262

#10 'survival' 626181

#11 'prognosis' 458131

#12 'mortality' 671502

#13 prognos* OR outcome* OR survival OR mortality

OR recurren* OR predit* 3091168

#14 #10 OR #11 OR #12 OR #13 3578470

#15 #6 AND #9 AND #14 482
