# Supplementary material for: Relationship Between the Use of Statins and Patient Survival in Colorectal Cancer: A Systematic Review and Meta-Analysis
Source: PLoS One. 2015 Jun 1;10(6):e0126944. doi: 10.1371/journal.pone.0126944 (PMC4451009; doi:10.1371/journal.pone.0126944)
Supplement: S1 Table — (DOCX) [file pone.0126944.s002.docx]

**Supporting Information**

**S1 Table.** Quality assessment of the included studies

| **Study** | **Q1** | **Q2** | **Q3** | **Q4** | **Q5** | **Q6** | **Q7** | **Q8** | **Score** |
| --- | --- | --- | --- | --- | --- | --- | --- | --- | --- |
| Cardwell/2014 | 0 | 1 | 1 | 1 | 2 | 1 | 1 | 0 | 7 |
| Lakha/2012  2012 | 0 | 1 | 1 | 1 | 2 | 1 | 1 | 0 | 7 |
| Mace/2013  2013 | 0 | 1 | 1 | 1 | 2 | 1 | 1 | 0 | 7 |
| Ma/2013 | 0 | 1 | 1 | 1 | 2 | 1 | 1 | 0 | 7 |
| Siddiqui/2009  2009 | 0 | 1 | 1 | 1 | 1 | 1 | 1 | 0 | 6 |
| Ng/2011  2011 | 0 | 1 | 1 | 1 | 1 | 1 | 1 | 0 | 6 |
| Nielsen/2012  2012  (Nationwide Study) | 0 | 1 | 1 | 1 | 2 | 1 | 1 | 1 | 8 |

**Questions for cohort study (one star is defined as one score)**

Q1: Representativeness of the exposed cohort

a) truly representative of the average CRC patient (describe) in the community (1 star)

b) somewhat representative of the average CRC patient in the community (1 star)

c) selected group of users eg. nurses, volunteers

d) no description of the derivation of the cohort

Q2: Selection of the non exposed cohort

a) drawn from the same community as the exposed cohort (1 star)

b) drawn from a different source

c) no description of the derivation of the non exposed cohort

Q3: Ascertainment of exposure

a) secure record (eg surgical records) (1 star) b) structured interview (1 star)

c) written self report d) no description

Q4: Demonstration that outcome of interest was not present at start of study

a) yes (1 star) b) no

Q5: Comparability of cohorts on the basis of the design or analysis

a) study controls for the most important factor (1 star)

b) study controls for any additional factor Outcome (1 star) (Age, stage, sex, etc.)

Q6: Assessment of outcome

a) independent blind assessment (1 star) b) record linkage (1 star)

c) self report d) no description

Q7: Was follow-up long enough for outcomes to occur

a) yes ( 2 years or all patients died or relapsed within 2 years) (1 star) b) no

Q8: Adequacy of follow up of cohorts

a) complete follow up - all subjects accounted for (1 star)

b) subjects lost to follow up unlikely to introduce bias - small number lost - > 20 % (select an adequate %) follow up, or description provided of those lost) (1 star)

c) follow up rate < 80% (select an adequate %) and no description of those lost

1. no statement
